# IMAGO: An Improved Model Based on Attention Mechanism for Enhanced Protein Function Prediction

**DOI:** 10.3390/biom15121667

**Published:** 2025-11-29

**Authors:** Meiling Liu, Longchang Liang, Qiutong Wang, Yunmeng Zhang, Lin Shi, Tianjiao Zhang, Zhenxing Wang

**Affiliations:** College of Computer and Control Engineering, Northeast Forestry University, Harbin 150040, China

**Keywords:** deep learning, transformer, attention mechanism, protein function prediction

## Abstract

Protein function prediction plays an important role in the field of biology. With the wide application of deep learning in the field of bioinformatics, more and more natural language processing (NLP) technologies are applied to the downstream tasks in the field of bioinformatics, and it has also shown excellent performance in protein function prediction. Protein-protein interaction (PPI) networks and other biological attributes contain rich information critical for annotating protein functions. However, existing deep learning networks still suffer from overfitting and noise issues, resulting in low accuracy in protein function prediction. Consequently, developing efficient models for protein function prediction remains a popular and challenging topic in the application of NLP in bioinformatics. In this study, we propose a novel protein function prediction model based on attention mechanisms, termed IMAGO. This model employs the Transformer pre-training process, integrating multi-head attention mechanisms and regularization techniques, and optimizes the loss function to effectively reduce overfitting and noise issues during training. It generates more robust embeddings, ultimately improving the accuracy of protein function prediction. Experimental results on *human* and *mouse* datasets indicate that our model surpasses other protein function prediction models across multiple metrics. Thus, this efficient, stable, and accurate deep learning model holds significant promise for protein function prediction.

## 1. Introduction

Proteins are the core molecules executing nearly all biological functions within living organisms. They play crucial roles in catalyzing reactions, transmitting signals, and providing structural support both inside and outside cells. Understanding protein functions is therefore vital for elucidating biological processes and developing new drugs [1]. The study of protein functions not only helps in comprehending the basic mechanisms of organisms but also provides important clues for drug development, disease diagnosis, and treatment. However, due to the high cost and time consumption of experimental methods, despite over 200 million known proteins, less than 1% of protein functions have been elucidated [2,3]. Experimentally determining all protein functions is nearly impossible in reality. Consequently, computational methods for predicting protein functions have emerged as an effective alternative. These methods infer protein functions primarily from protein sequences, structures, and other biological information. This not only accelerates the discovery of unknown protein functions but also provides critical clues for new drug development, disease diagnosis, and treatment [4].

Protein functions are typically classified into three aspects by the Gene Ontology (GO): Biological Process (BP), Molecular Function (MF), and Cellular Component (CC) [2]. In recent years, with the development of deep learning technologies, the accuracy and efficiency of protein function prediction have significantly improved. Deep learning methods, leveraging large biological datasets, can automatically extract features and perform complex pattern recognition, making large-scale function prediction feasible [5]. Therefore, combining experimental data with computational predictions can more comprehensively and efficiently reveal protein functions, providing a crucial foundation for biomedical research and clinical applications.

In recent years, the field of protein function prediction has advanced rapidly, resulting in numerous automated protein function annotation algorithms [6]. Notably, multimodal fusion approaches based on deep learning, including DeepGO-SE [7], GPSFun [8], and TAWFN [9], have further advanced the prediction accuracy and applicability of protein function annotation. Protein function prediction methods have become increasingly diverse, generally classified into four categories: sequence similarity methods, structure similarity methods, network-based machine learning methods, and hybrid source methods.

Sequence similarity methods were among the earliest approaches for protein function prediction, based on the sequence-function homology hypothesis [10,11,12] that proteins with high sequence similarity often share similar functions. Blakeley et al. provided an overview of sequence alignment and homology analysis methods, noting their limitations in cases where homologous sequences are scarce in databases [13]. Deng et al. introduced a sequence encoding method using LSTM networks, significantly improving prediction accuracy [14]. However, studies have shown that some proteins may have similar functions despite having low sequence similarity [10,15], posing challenges to sequence-based methods.

Structure-based methods predict protein functions by utilizing the three-dimensional structures of proteins [16]. Structure similarity methods focus on protein three-dimensional structures, with the premise that proteins with similar structures tend to exhibit similar functions [17]. Gomez et al. developed a function prediction framework based on structural alignment [9]. Liu et al. proposed a framework using a multilevel attention mechanism to realize the deep interactive fusion of protein sequence embedding and structural features extracted from contact graphs [18]. Additionally, structure similarity prediction has benefited from convolutional neural network (CNN) models, which automatically capture functional motifs in local protein structures, effectively enhancing prediction accuracy [19]. However, this prediction method may be inaccurate in some cases [17].

Network-based machine learning methods utilize protein-protein interaction (PPI) networks or gene co-expression networks to build predictive models [20]. Mostafavi et al. predicted protein function through label propagation between network nodes [21]. Wang et al. proposed a framework based on graph neural networks (GNNs) that effectively captures complex interactions between proteins [22]. Compared to traditional network analysis methods, GNNs propagate information across network nodes and edges, further enhancing prediction performance. However, the application of these methods can be limited by several factors, including inherent noise in PPI networks, the computational complexity of processing large-scale biological networks, and the expertise required for effective feature engineering and model tuning [23].

Finally, hybrid source methods integrate multiple sources of information, including sequence, structure, and PPI networks, to provide a more comprehensive function prediction. These methods leverage the diversity of biological data to capture richer functional features. Wu et al. proposed a multimodal data fusion model that combines different data sources to achieve higher prediction accuracy [24]. Hybrid source methods are also regarded as a promising direction for future protein function prediction, addressing the information limitations associated with single-source data [17]. This method has many applications in other biological fields, such as RNA prediction. Zhang et al. uses a variety of biological signals, such as histone modification and RNA-seq data, which significantly improves the accuracy of eRNA recognition [25]. In the field of protein function prediction, the model [26] proposed by Wu et al., which combines PPI network with protein’s biological attributes, has shown remarkable effects, but they are also affected by noise and details of over-fitting in training data. Therefore, there is an urgent need for new methods to improve attention mechanism-based models to enhance the accuracy of protein function prediction.

In this study, we proposed a new protein function prediction method, IMAGO, based on attention mechanism. In the pre-training process using Transformer, regularization technology was added, and the loss function was optimized. Comparative experiments on *human* and *mouse* datasets demonstrated that the IMAGO model improved the accuracy of protein function prediction by up to 10.65%, 11.76%, 9%, 38.89%, and 10.85% in m-AUPR, m-AUPR, F1, ACC, and Fmax, respectively, suggesting that our model achieves a modest improvement in protein function prediction accuracy under specific datasets and conditions.

## 2. Materials and Methods

### 2.1. Dataset

Our dataset includes two species: *human* (taxonomy code 9606) and *mouse* (taxonomy code 10090). PPI data and protein sequence data were retrieved from the STRING database (v11.5) [1]. We used “combined” type PPI data comprising “experimental”, “coexpression”, “cooccurrence”, “neighborhood”, “fusion”, “database”, and “text mining” types. GO functional annotation data were retrieved from the Gene Ontology resource website (version 2022-01-13). Protein attributes, including subcellular localization and Pfam protein domain annotations, were obtained from the Uniprot database (v3.5.175). Proteins annotated before 1 January 2018, were defined as the training set, those annotated between 2 January 2018, and 31 December 2020, were defined as the validation set, and those annotated after 1 January 2021, were defined as the test set. The partitioning was based on the earliest recorded GO annotation date for each protein to ensure exclusive assignment to one dataset. For proteins with multiple annotations at different time points, the earliest date determined their assignment to the training, validation, or test set. To ensure sufficient proteins for each label, we used GO terms with at least 10, 5, and 1 proteins in the training, validation, and test sets, respectively. Additionally, to reduce the influence of dependencies between GO terms, we removed GO terms that annotated more than 5% of the PPI network proteins of the species. The statistics of the training, validation, and test sets for *human* and *mouse* data used in this study are shown in Table 1.

It is crucial to note that the data partitioning between the pre-training and fine-tuning stages in this study strictly follows a chronological order. The protein samples utilized during the self-supervised pre-training phase are exclusively drawn from the training set (i.e., proteins annotated before 1 January 2018). The information from proteins in the validation and test sets remains entirely inaccessible to the model during pre-training. This protocol ensures that the model is not exposed to data from future time points during its initial representation learning, thereby effectively mitigating potential data leakage. In the subsequent fine-tuning stage, the model is trained solely using the labels from the training set. The validation set is employed for strategies such as early stopping to prevent overfitting, and the final performance is evaluated rigorously on the completely independent test set.

### 2.2. Integration of PPI and Protein Attributes

The IMAGO model employs a multi-head attention mechanism to integrate single-species PPI networks and protein biological attributes. Based on the previously published transformer-based autoencoder model (TransformerAE) [26], this model takes protein attributes and PPI networks as inputs and outputs low-dimensional embeddings. The PPI network is transformed into a weighted adjacency matrix, with weights determined by the minimum normalized scores of the corresponding evidence. Protein attributes, including Pfam domains and subcellular locations, are encoded into binary vectors using a bag-of-words model: if a protein possesses a specific domain or subcellular location, the corresponding element in the vector is set to 1. To enhance data quality, domain terms occurring less than six times in proteins were removed.

**Figure 1:**
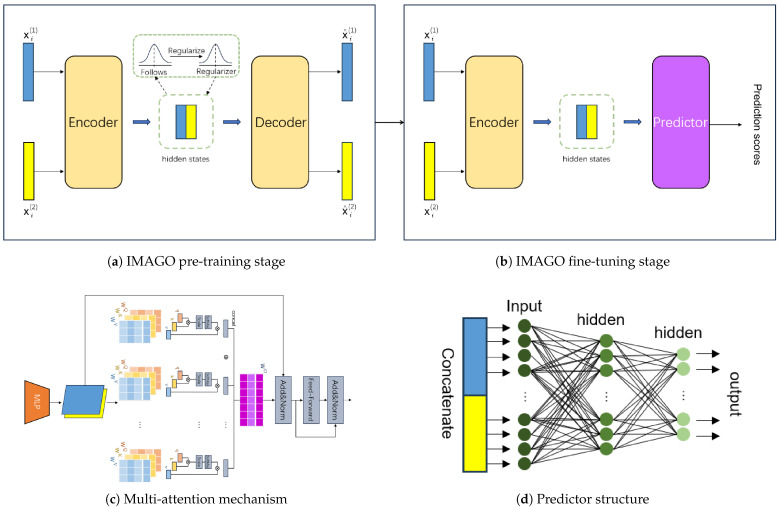
IMAGO model diagram. (**a**) IMAGO pre-training stage: the model uses the training set proteins to reconstruct their original features (PPI network and attributes). (**b**) IMAGO fine-tuning stage: the pre-trained encoder is frozen or jointly fine-tuned with a new predictor using the training set(with GO labels), and the validation set is used for hyperparameter tuning. (**c**) Multi-attention mechanism. (**d**) Predictor structure. Where $x_{i}^{\left(1\right)}$ and $x_{i}^{\left(2\right)}$ are the original features of protein *i* from different sources, and ${\hat{x}}_{i}^{\left(1\right)}$ and ${\hat{x}}_{i}^{\left(2\right)}$ are the reconstructed protein *i* features.

The IMAGO model comprises two stages: self-supervised pre-training and fine-tuning (see Figure 1a,b).

### 2.3. Self-Supervised Pre-Training

The IMAGO model adopts an encoder–regularizer–decoder structure to learn the hidden embedding vectors of proteins by reconstructing the original features. During pre-training, the encoder and decoder are used to integrate information from the two data sources. For protein *i*, its two original source features are represented as $x_{i}^{\left(1\right)}\in R^{d(1)}$ and $x_{i}^{\left(2\right)}\in R^{d(2)}$, where d(m) is the feature dimension of source *m*.

The encoder has two parallel multi-layer perceptrons (MLPs), each used for one source feature, and *L* multiple multi-head attention layers. To match the original data dimensions, the original feature vector of protein *i* from source *m*, $x_{i}^{\left(m\right)}$ is projected to a common vector with *d*, dimensions by a two-layer MLP, which is defined as:

(1) $$\mathrm{MLP}\left(x\right)=f(\mathrm{LN}\left(W_{2}f\left(\mathrm{LN}\left(W_{1}x+b_{1}\right)\right)\right)+b_{2})$$

where *f* is the non-linear activation function, LN is the layer normalization function [23], and $W_{1}\in R^{d\left(m\right)*d_{e}}$, $W_{2}\in R^{d_{e}*d}$ are the weight matrices, and $b_{1}\in R^{d_{e}}$, $b_{2}\in R^{d}$ are the bias vectors, $d_{e}$ is the size of the MLP hidden layer. Then, the projected vectors from the two sources are cross-integrated through multi-head attention layers to generate hidden protein embedding vectors.

In the TransformerAE model, the adjacency matrix and protein attribute matrix jointly pass through the encoder’s six-layer multi-head attention layers (see Figure 1c) and the decoder’s six-layer multi-head attention layers to achieve information fusion from both data sources. The attention mechanism’s formula is:

(2) $$attention\left(Q,K,V\right)=softmax\left(\frac{QK^{T}}{\sqrt{d_{k}}}\right)V$$

where *Q* is the query, *K* is the key, and *V* is the value matrix [27], and $d_{k}$ is the dimension of the query and key vectors in the matrix.

The regularizer’s primary role is to adjust and optimize feature representations in the network, making these features more conducive to the model’s generalization ability. In deep learning models, regularization helps the model maintain robustness when faced with unseen data, preventing the model from overfitting specific noise and details in the training data. By processing intermediate features in the network to adjust their distributions, the regularizer can reduce differences between features and increase their intrinsic consistency. This typically helps improve classification or other task accuracies. In the pre-training process, the regularizer also continuously updates. The structure of the regularizer is as follows:

(3) $$R_{\theta }\left(Z\right)=W_{R3}\sigma \left(W_{R2}\left(\sigma \left(W_{R1}Z\right)\right)\right)$$

where $W_{R1}$ and $W_{R2}$ are the weights of the hidden layers, while $W_{R3}$ is the parameter in the output layer, and σ is the sigmoid activation function. Updating of regularizer in pre-training:

(4) $$\begin{array}{cc} Loss_{reg} & =\sum_{i=1}^{N}\sum_{m=1}^{2}\left(f\left(h_{i}^{\left(m\right)}\right)-f\left(r^{\left(m\right)}\right)\right) \end{array}$$

where *N* is the total number of proteins in the PPI network, *f* is a regularization function, $h_{i}^{\left(m\right)}$ is the embedding of the hidden protein of the source m, and $r^{\left(m\right)}$ is a random vector with normal distribution randomly generated from it, and its shape is the same as the embedding vector of the hidden protein. The regularizer is constantly updated in the pre-training process, and its features conform to the specific expected distribution by adjusting the distribution of input vectors. The random normal distribution vectors act as the target distribution, guiding the input vectors’ distribution to gradually approach a normal distribution.

The decoder’s structure mirrors the encoder’s. The decoder first inputs the hidden embedding vectors into *L* multi-head attention layers.Then, for protein *i*, the feature vector of source *m* is reconstructed by an MLP whose structure is symmetric to the corresponding MLP in encoder, denoting as ${\hat{x}}_{i}^{\left(m\right)}$. The decoder uses the function σ as the activation function of the MLP output layer.

During self-supervised pre-training, we optimized the loss function to minimize the difference between the original inputs before entering the encoder and the reconstructed outputs after passing through the decoder. Here, we present the loss function’s expression, starting with the binary cross-entropy loss:

(5) $$Loss_{BCE}=\frac{1}{N}\sum_{i=1}^{N}\sum_{m=1}^{2}\sum_{j=1}^{d(m)}-\left[x_{ij}^{\left(m\right)}\log{\hat{x}}_{ij}^{\left(m\right)}+\left(1-x_{ij}^{\left(m\right)}\right)\log\left(1-{\hat{x}}_{ij}^{\left(m\right)}\right)\right]$$

where *N* is the total number of proteins in the PPI network, $x_{ij}^{\left(m\right)}$ and ${\hat{x}}_{ij}^{\left(m\right)}$ are the *j* dimensions of $x_{i}^{\left(m\right)}$ and ${\hat{x}}_{i}^{\left(m\right)}$, respectively. In addition, constraints are added to the embedding generated by the encoder to regularize the model. The expression of the regularization term is as follows:

(6) $$\begin{array}{cc} Loss_{regular} & =\sum_{i=1}^{N}\sum_{m=1}^{2}f\left(h_{i}^{\left(m\right)}\right) \end{array}$$

where *f* is defined as the L2 regularization function, given by $f\left(x\right)=\lambda {∥x∥}_{2}^{2}$, with λ denoting the regularization coefficient. We also introduce cosine similarity loss, which is used to measure the cosine similarity between the original embedding and the reconstructed embedding to ensure that the embedding is similar to the original embedding in direction. The expression is as follows:

(7) $$\begin{array}{cc} Loss_{cosine} & =\sum_{i=1}^{N}\sum_{m=1}^{2}\frac{1-(\frac{h_{i}^{\left(m\right)}\cdot x_{i}^{\left(m\right)}}{∥h_{i}^{\left(m\right)}∥∥x_{i}^{\left(m\right)}∥})}{h_{i}^{\left(m\right)}} \end{array}$$

where *N* is the total number of proteins in the PPI network, $h_{i}^{\left(m\right)}$ is the embedding of the hidden protein of the source m, $x_{i}^{\left(m\right)}$ is the original feature vector of source m.

The loss function is computed as follows:

(8) $$\begin{array}{cc} Loss & =Loss_{BCE}+Loss_{regular}+Loss_{consine} \end{array}$$

Each part of the loss plays a crucial role in ensuring the model effectively learns and maintains the integrity of the learned embeddings during training.

### 2.4. Fine-Tuning for Protein Function Prediction

In this study, protein function prediction is modeled as a multi-label task. We combined the pre-trained encoder with a predictor to predict protein functions. The predictor is a two-layer perceptron used to predict protein labels (see Figure 1d). Given that the number of target GO terms is *K*, the predictor takes the concatenation of the embeddings from the two sources $h_{i}^{\left(1\right)}$ and $h_{i}^{\left(2\right)}$ generated by the encoder as input and outputs a score vector of dimension *K* for the GO terms. The prediction score vector ${\left[p_{i1},\ldots p_{iK}\right]}^{T}$ of protein *i* is defined as follows:

(9) $${\left[p_{i1},\ldots p_{iK}\right]}^{T}=\sigma \left(W_{o}\sigma \left(W_{b}\left(∥h_{i}^{\left(m\right)}\right)+b_{b}\right)+b_{o}\right)$$

where ∥ is concatenation operator, and σ is the sigmoid function, $d_{h}$ is the size of the predictor’s hidden layer, $W_{b}\in R^{2d*d_{h}}$ and $W_{o}\in R^{d_{h}*K}$ are the weight matrices of predictor’s hidden and output layers, respectively. $b_{h}\in R^{d_{h}}$ and $b_{o}\in R^{K}$ are the bias vectors of predictor’s hidden and output layers, respectively. For protein function prediction, we used the Asymmetric Loss (ASL) [28] as the loss function to achieve better performance in the multi-label task. ASL is designed to address the potential severe imbalance of samples between labels in typical multi-label datasets. ASL is defined as follows:

(10) $$ASL=\frac{1}{N_{train}\cdot K}\sum_{i=1}^{N_{train}}\sum_{k=1}^{K}\left[-y_{ik}{\left(1-p_{ik}\right)}^{\gamma_{+}}\log\left(p_{ik}\right)-\left(1-y_{ik}\right){\left(p_{ik}\right)}^{\gamma_{-}}\log\left(1-p_{ik}\right)\right]$$

where $N_{train}$ is the number of proteins in the training set, *K* is the number of functions in a specific category, $\gamma_{+}$ and $\gamma_{-}$ are the focusing parameters for positive and negative samples. In this study, we set $\gamma_{+}$ to 0 and $\gamma_{-}$ to 2 to reduce the contribution of easy negative samples, encouraging the model to make more positive predictions.

### 2.5. Experimental Setup

We used the same empirical hyperparameter set for training on the three GO aspects (BP, MF, and CC) of the two species (*human* and *mouse*), and use the verification set and training set to train the model. The performance on the test set will be the evaluation result. In addition, we also carried out experiments on fruit flies and *zebrafish* to verify our model. The pre-training and fine-tuning parameters of the model, except for the pre-training epochs, are consistent with the CFAGO model [26]. This is because according to the research [6], the effect of 500 rounds of pre-training with Transform AE is basically the same as that of 5000 rounds. The regularizer comprises an MLP with an input dimension of 512, hidden layer dimensions of 64 and 32, and an output dimension of 1. The IMAGO model was implemented in Python 3.11 using Pytorch 2.1, and a single training session on an RTX 4060 Ti GPU with 16GB of memory took approximately 5 to 6 h.

For the visualization of protein embedding clusters, we employed t-Distributed Stochastic Neighbor Embedding (t-SNE), a nonlinear dimensionality reduction technique particularly effective for visualizing high-dimensional data by preserving local structures. Implementations used the scikit-learn TSNE class with default parameters, reducing embeddings to two dimensions (random state = 0) to generate the two-dimensional projections.

We compare IMAGO with two baseline methods and five network-based models. The naive method directly assigns the relative frequency of terms in the training set to each protein in the test set as a predictive score. Based on the sequence similarity, the BLAST method transfers the GO term of the protein in the training set to the corresponding protein in the test set through blastp and assigns it according to the comparison score. GeneMANIA [21] predicts gene function through a weighted proximity algorithm, and integrates multiple data sources into a unified network to dynamically adjust the weight of each data source to optimize the prediction effect. Mashup [7] generates a balanced hidden diffusion state through multi-network topology fusion, using different PPI network information. Based on MLPAE, deepNF [8] embeds multiple PPI networks into a joint potential representation. Graph2GO [9] combines variational graph automatic encoder (GAE) and sequence similarity network to integrate protein attributes with PPI network features for function prediction. CFAGO [26] uses the Transformer-based automatic encoder (TransformerAE) to cross-fuse the PPI network and protein attributes.

### 2.6. Evaluation Metrics

In this study, we used five metrics to evaluate prediction performance, including two types of area under the precision-recall curve, namely micro-average AUPR (m-AUPR) and macro-average AUPR (M-AUPR), F1 score (F1), accuracy (ACC), and F-max score (Fmax). The first three metrics are function-centric, evaluating proteins annotated with each GO term, while the latter two metrics are protein-centric, assessing GO terms annotated on each protein. *Fmax* is defined as follows:

(11) $$Fmax=\underset{\tau }{max}\left\{\frac{2*precision(\tau )*recall(\tau )}{precision(\tau )+recall(\tau )}\right\}$$

where τ is a flexible threshold for obtaining the highest Fmax score.

The precision and recall for multi-label tasks are defined as follows:

(12) $$\left\{\begin{array}{c} precision\left(\tau \right)=\frac{1}{s(\tau )}\sum_{i=1}^{s(\tau )}\frac{\sum_{k}I\left(p_{ik}>\tau \wedge y_{ik}\equiv 1\right)}{\sum_{c}I\left(p_{ik}>\tau \right)}\\ recall\left(\tau \right)=\frac{1}{n}\sum_{i=1}^{n}\frac{\sum_{c}I\left(p_{ik}>\tau \wedge y_{ik}\equiv 1\right)}{\sum_{c}I\left(y_{ik}\equiv 1\right)} \end{array}\right.$$

s(τ) denotes the number of proteins that are predicted with at least one function. *k* is the total number of labels for a specific functional category. $p_{ik}$ is the predicted score for the function and $y_{ik}$ is the ground truth with 1 indicating the existence of the function. *n* is the total number of proteins to be evaluated.

## 3. Results

### 3.1. Experimental Results

**Figure 2:**
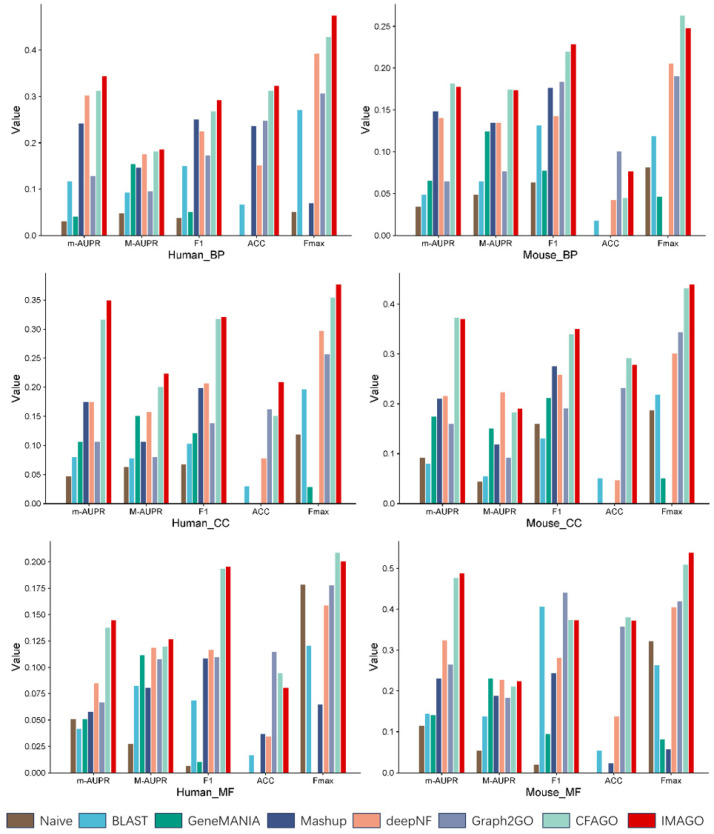
Performance of IMAGO and other models in protein function prediction.

Figure 2 shows that IMAGO is superior to other models in GO and many indicators on *human* and *mouse* data sets. Among them, IMAGO improves the *human* data set most obviously. Compared with the CFAGO model, the highest improvements in m-AUPR, m-AUPR, F1, ACC, and Fmax are 10.65%, 11.76%, 9%, 38.89% and 10.85%. And the improvements in the *human* dataset are most notable.The comparison results between IMAGO and other models indicate that IMAGO, with the addition of regularization techniques and optimized loss functions, achieves the highest scores in most cases for protein function prediction.

**Figure 3:**
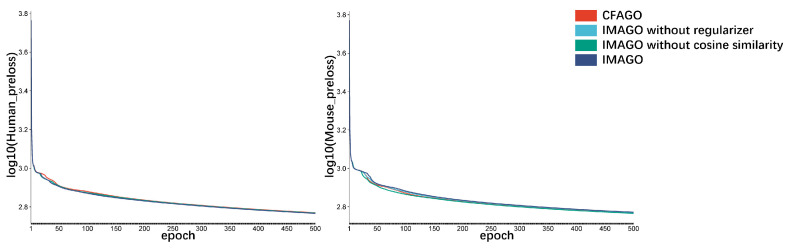
Loss in the pre-training stage of the model. On the left is the pre-training loss of *human* data set, and on the right is the pre-training loss of *mouse* data set.

The performance of IMAGO in the *mouse* data set is not as good as that in the *human* data set, which may be related to the characteristics of the data set. It can also be reflected from the loss in the pre-training stage of IMAGO (see Figure 3), and the convergence speed of the *mouse* data set is slower than that of CFAGO. It is precisely because different data sets have different noise levels and feature distributions that the regularization and loss function optimization effect of IMAGO on some data sets may be more advantageous.

### 3.2. Ablation Experiment

We conducted ablation experiments on *human* datasets to investigate the contribution of training epochs, the regularizer, and the optimized loss function to performance improvement. Among them, the indicator m-AUPR is shown in Table 2.

We set up five control groups: CFAGO with 500 training epochs (equivalent to IMAGO without the regularizer and optimized loss function), IMAGO with 5000 training epochs, IMAGO with 500 training epochs without the regularizer, IMAGO with 500 training epochs without the optimized loss function, and IMAGO with 500 training epochs. We observed that IMAGO with 500 training epochs and IMAGO with 5000 training epochs achieved the highest scores in different GO aspects. This is attributed to the cooperation between the regularizer and the loss function, which together suppress the long tail noise and modal conflict in biological data and make the model perform best in complex GO categories (such as *human* BP).

### 3.3. Visualization of Protein Embeddings

**Figure 4:**
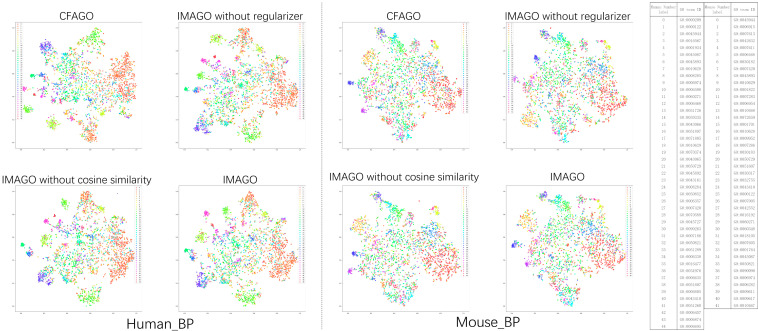
Evaluation of clustering visualization in BP (visualized using t-SNE dimensionality reduction technique).

To intuitively demonstrate the quality of protein representations learned by the IMAGO model, we employed t-SNE (t-distributed Stochastic Neighbor Embedding) to project the generated protein embeddings into a two-dimensional space for visualization. As illustrated in Figure 4, which uses the Biological Process (BP) category of the *human* dataset as an example, distinct colors represent proteins annotated with specific GO functional terms. The visualization reveals that protein embeddings with similar functions form well-defined clusters in the projected space, while those with divergent functions are clearly separated. This pattern demonstrates that the IMAGO model effectively captures the underlying semantic relationships between protein functions, producing highly discriminative embedding representations. These qualitative results provide intuitive evidence supporting the model’s superior classification performance, as quantitatively reported in the previous sections.

The exceptional capability of IMAGO to differentiate protein representations can be attributed to the synergistic effects of its core components: the multi-head attention mechanism adeptly captures long-range dependencies and complex interaction patterns among proteins; the incorporated regularization techniques mitigate overfitting during training, thereby enhancing the robustness of the generated embeddings; and the optimized loss function ensures the effective learning and preservation of embedding integrity throughout the training process. Collectively, these techniques empower IMAGO to produce highly distinguishable and robust protein embeddings, which form a solid foundation for accurate protein function prediction.

### 3.4. Comparative Evaluation of IMAGO on Diverse Species

In addition, in order to verify the effect of the IMAGO model on other species, we carried out experiments on *Drosophila* and *Zebrafish* according to the previous method of data set processing, and the experimental results are shown in Table 3.

The performance of the IMAGO model in cross-species testing is highly dependent on biological data types and species characteristics: molecular function (MF) is predicted to reach the dominant performance in *zebrafish*, which is due to the evolutionary conservation and complete annotation of enzyme active sites, while biological process (BP) is predicted to collapse in *zebrafish* due to the space-time specific labeling gap in the development process; *Human* are ahead of BP by improving the pathway database, but the complex regulatory mechanism leads to the weakness of MF. The prediction of subcellular localization (CC) shows the cross-species stability due to the structural conservatism of organelles, revealing the fundamental contradiction that the prediction of static structural characteristics is significantly better than that of dynamic biological processes, and the performance of the model is limited by the annotation completeness and functional conservatism of species data [29].

**Figure 5:**
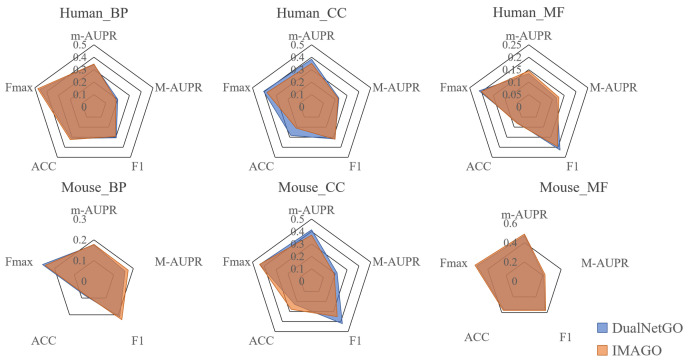
Performance Comparison of IMAGO and DualNetGO on *Human* and *Mouse* Datasets across Gene Ontology Aspects.

To further position IMAGO against a contemporary state-of-the-art model that addresses the same task with a complementary approach, we compare our results with DualNetGO [6], another advanced model built upon the CFAGO framework (see Figure 5). While both models aim to enhance protein function prediction, their improvement strategies differ: IMAGO introduces internal regularization and optimizes the loss function to learn more robust embeddings, whereas DualNetGO employs a dual-network architecture to intelligently select features from multiple PPI network embeddings.

A direct comparison of the *human* and *mouse* datasets(as these are common to both studies) reveals the respective strengths of each approach. For instance, on the *Human* BP dataset, IMAGO achieves an Fmax of 0.475, while DualNetGO reports an Fmax of 0.459 (as per their publication). This suggests that IMAGO’s regularization techniques effectively capture broader functional patterns. Conversely, on the *Mouse* MF dataset, DualNetGO might show superior performance, highlighting the advantage of its feature selection mechanism when dealing with specific functional categories where certain PPI evidence types are particularly informative. This comparative analysis underscores that the choice of an optimal model may be context-dependent, influenced by the target species and the specific GO aspect (BP, MF, CC). IMAGO’s strength lies in its generalized robustness against noise and overfitting, making it particularly suitable for scenarios with complex and noisy PPI data.

## 4. Discussion

The superior performance of IMAGO over CFAGO, particularly on *human* datasets, can be attributed to the synergistic effects of three core improvements: Regularization Counteracts Overfitting: The CFAGO model is prone to overfitting training data noise and specific details, limiting its generalization on test sets. IMAGO addresses this by incorporating a regularizer that constrains the distribution of hidden layer embeddings to approximate a normal distribution. This smoothing of learned feature representations reduces the model’s reliance on incidental patterns in training data, thereby enhancing robustness; Loss Function Optimization Ensures Feature Integrity: IMAGO introduces cosine similarity loss during pre-training, which ensures that the encoder-generated embedding vectors align directionally with the original input features, rather than merely achieving numerical reconstruction. This preserves more essential semantic information from the biological data, providing higher-quality features for downstream function prediction tasks; Component Synergy: The multi-head attention mechanism captures complex protein-protein interactions; the regularizer ensures these patterns are generalizable rather than specific; and the optimized loss function maintains fidelity in the encoding-decoding process. Together, these components enable IMAGO to learn more discriminative and generalizable protein representations, leading to improved performance in function prediction.

## 5. Conclusions

In this study, we propose IMAGO, a deep learning model leveraging attention mechanisms for protein function prediction. IMAGO integrates multi-source biological data within a single species through an encoder-regularizer-decoder architecture, where pre-training learns generalized protein representations and fine-tuning optimizes function prediction. Experimental validation demonstrates IMAGO’s superior performance on *human* and *mouse* datasets. Future work will focus on three critical directions revealed by cross-species validation: Developing spatiotemporal-aware attention mechanisms to model dynamic biological processes where current performance lags. Implementing adaptive regularization techniques that address species-specific data imbalances, particularly for long-tail GO term distributions. Establishing cross-species transfer learning frameworks to enhance prediction in less-studied organisms by leveraging conserved functional knowledge. These advancements will strengthen IMAGO’s capability to decipher disease mechanisms and accelerate drug target discovery across diverse species.

## Figures and Tables

**Table 1:** The data statistics of *human* and *mouse* branches of gene ontology were used in the study, the same as in [26].

Species	Statistics	BP	MF	CC
*Human*	#GO terms	45	38	35
	#training proteins	3197	2747	5263
	#validation proteins	304	503	577
	#testing proteins	182	719	119
*Mouse*	#GO terms	42	17	37
	#training proteins	2714	1185	4014
	#validation proteins	336	232	694
	#testing proteins	155	126	147

BP (Biological Process) describes broader biological objectives accomplished by multiple molecular activities; MF (Molecular Function) defines the specific biochemical activities of a protein at the molecular level; CC (Cellular Component) indicates the locations in a cell where a protein is active.

**Table 2:** Evaluation of ablation regularization and optimized loss function in m-AUPR (*human* dataset). Underline indicates the highest score.

Model	Regularizer	Loss	Epoch	BP	MF	CC
CFAGO	No	No	500	0.313	0.138	0.317
IMAGO	Yes	Yes	5000	0.312	0.147	0.306
IMAGO	No	Yes	500	0.326	0.144	0.303
IMAGO	Yes	No	500	0.310	0.145	0.329
IMAGO	Yes	Yes	500	0.346	0.145	0.350

**Table 3:** Experimental results of IMAGO model on datasets of four different species across BP, MF and CC aspects.

Biological Process (BP) Aspect					
Species	m-AUPR	M-AUPR	F1	ACC	Fmax
*Human*	0.345	0.187	0.293	0.324	0.475
*Mouse*	0.178	0.174	0.229	0.077	0.248
*Drosophila*	0.309	0.171	0.273	0.217	0.442
*Zebrafish*	0.054	0.068	0.068	0.010	0.121
**Molecular Function (MF) Aspect**					
Species	m-AUPR	M-AUPR	F1	ACC	Fmax
*Human*	0.145	0.127	0.196	0.081	0.201
*Mouse*	0.489	0.224	0.374	0.373	0.539
*Drosophila*	0.350	0.340	0.339	0.226	0.441
*Zebrafish*	0.934	0.389	0.526	0.878	0.911
**Cellular Component (CC) Aspect**					
Species	m-AUPR	M-AUPR	F1	ACC	Fmax
*Human*	0.350	0.224	0.321	0.210	0.377
*Mouse*	0.371	0.191	0.351	0.279	0.440
*Drosophila*	0.162	0.269	0.272	0.079	0.289
*Zebrafish*	0.385	0.440	0.507	0.118	0.604

## Data Availability

The original data presented in the study are openly available in github at https://github.com/Lianglongchang/IMAGO (accessed on 20 January 2025).

## References

[1] Szklarczyk D., Kirsch R., Koutrouli M., Nastou K., Mehryary F., Hachilif R., Gable A.L., Fang T., Doncheva N.T., Pyysalo S. (2023). The STRING database in 2023: Protein-protein association networks and functional enrichment analyses for a wide range of organisms. Nucleic Acids Res..

[2] Aleksander S., Balhoff J., Carbon S., Cherry J., Drabkin H., Ebert D., Feuermann M., Gaudet P., Harris N. (2023). The Gene Ontology Knowledgebase in 2023. Genetics.

[3] UniProt Consortium (2023). UniProt: The Universal Protein Knowledgebase. Nucleic Acids Res..

[4] Jones D.T., Thornton J.M. (2021). The Impact of Bioinformatics on Biological Research. Current Opinion in Structural Biology.

[5] Lai B., Xu J. (2022). Accurate Protein Function Prediction via Graph Attention Networks with Predicted Structure Information. Briefings Bioinf..

[6] Chen Z., Luo Q. (2023). DualNetGO: A Dual Network Model for Protein Function Prediction via Effective Feature Selection. Bioinformatics.

[7] Cho H., Berger B., Peng J. (2016). Compact Integration of Multi-Network Topology for Functional Analysis of Genes. Cell Syst..

[8] Gligorijević V., Barot M., Bonneau R. (2018). deepNF: Deep Network Fusion for Protein Function Prediction. Bioinformatics.

[9] Gomez J., Bonet J., Borràs C., Ferrer A. (2017). Structure-Based Function Prediction Using Structural Alignments and Clustering. Struct. Biol..

[10] Gligorijević V., Renfrew P.D., Kosciolek T., Leman J.K., Berenberg D., Vatanen T., Chandler C., Taylor B.C., Fisk I.M., Vlamakis H. (2021). Structure-Based Function Prediction Using Graph Convolutional Networks. Nat. Commun..

[11] Wang S., You R., Liu Y., Xiong Y., Zhu S. (2020). Graph Neural Networks for Protein Function Prediction. Nat. Commun..

[12] Chen B., Cheng X., Li P., Geng Y., Gong J., Li S., Bei Z., Tan X., Wang B., Zeng X. (2024). xTrimoPGLM: Unified 100-Billion-Parameter Pretrained Transformer for Deciphering the Language of Protein. Nat. Methods.

[13] Blakeley K.J., Harbison C.T., Parker C.T., Cline M.S., Smith J.R. (2015). Homology-Based Methods for Protein Function Prediction. Bioinform. J..

[14] Deng J., Guo J., Wang Z., Liu Y., Chen J., Hu X. (2018). Sequence Encoding via LSTM Networks for Protein Function Prediction. J. Comput. Biol..

[15] Meng L., Wang X. (2024). TAWFN: A Deep Learning Framework for Protein Function Prediction. Bioinformatics.

[16] Abramson J., Adler J., Dunger J., Evans R., Green T., Pritzel A., Ronneberger O., Willmore L., Ballard A.J., Bambrick J. (2024). Accurate Structure Prediction of Biomolecular Interactions with AlphaFold 3. Nature.

[17] Jumper J., Evans R., Pritzel A., Green T., Figurnov M., Ronneberger O., Tunyasuvunakool K., Bates R., Žídek A., Potapenko A. (2021). Highly Accurate Protein Structure Prediction with AlphaFold. Nature.

[18] Liu M., Wang S., Luo Z. (2025). Multistage Attention-Based Extraction and Fusion of Protein Sequence and Structural Features for Protein Function Prediction. Bioinformatics.

[19] Zhang Y., Wang Y., Liu X., Chen L. (2019). Applying CNN for Protein Structure Function Prediction. Deep Learn. Bioinf..

[20] Gu Z., Luo X., Chen J. (2023). Hierarchical Graph Transformer with Contrastive Learning for Protein Function Prediction. Bioinformatics.

[21] Mostafavi S., Ray D., Warde-Farley D., Grouios C., Morris Q. (2008). GeneMANIA: A Real-Time Multiple Association Network Integration Algorithm for Predicting Gene Function. Genome Biol..

[22] Vaswani A., Shazeer N., Parmar N., Uszkoreit J., Jones L., Gomez A.N., Kaiser Ł., Polosukhin I. (2017). Attention Is All You Need. Advances in Neural Information Processing Systems 30.

[23] Ba J.L., Kiros J.R., Hinton G.E. (2016). Layer Normalization. arXiv.

[24] Wu Z., Zhang S., Huang X., Li Y. (2021). Integrative Approaches for Protein Function Prediction. Multimodal Data Fusion in Bioinformatics.

[25] Fan K., Guan Y., Zhang Y. (2020). Graph2GO: A Multi-Modal Attributed Network Embedding Method for Inferring Protein Functions. GigaScience.

[26] Wu Z., Guo M., Jin X., Chen J., Liu B. (2023). CFAGO: Cross-Fusion of Network and Attributes Based on Attention Mechanism for Protein Function Prediction. Bioinformatics.

[27] Ridnik T., Ben-Baruch E., Zamir N., Noy A., Friedman I., Protter M., Zelnik-Manor L. Asymmetric Loss for Multi-Label Classification. Proceedings of the IEEE/CVF International Conference on Computer Vision (ICCV).

[28] Wang B., Geng Y., Cheng X. (2025). ProtGO: Universal Protein Function Prediction Utilizing Multi-Modal Gene Ontology Knowledge. Bioinformatics.

[29] Wang W., Shuai Y., Zeng M. (2025). DPFunc: Accurately Predicting Protein Function via Deep Learning with Domain-Guided Structure Information. Nat. Commun..

